# Dry Eye in Post-menopausal Women Visiting Community Eye Centre: An Observational Study

**DOI:** 10.31729/jnma.9202

**Published:** 2025-08-31

**Authors:** Kripa Joshi, Manish Poudel, Niraj Man Shrestha

**Affiliations:** 1 Department of Ophthalmology, Tilganga Institute of Ophthalmology, Kathmandu, Nepal; 2 Department of Statistics, Tilganga Institute of Ophthalmology, Kathmandu, Nepal; 3 Department of Orthopaedics, Manmohan Memorial Medical College and Teaching Hospital, Swayambhu, Nepal

**Keywords:** *dry eye*, *postmenopausal period*, *Schirmer's test*, *visual impairment*

## Abstract

**Introduction:**

Dry eye disease is one of the common disorders that may lead to severe ocular complications, visual impairment or even blindness and sometimes may be a manifestation of underlying systemic disease. Postmenopausal women are one of the most common groups to be affected by dry eye. The aim of the study was to assess the prevalence of dry eye in postmenopausal women and to describe its distribution according to age and duration of menopause.

**Methods:**

This cross-sectional study was conducted in a community eye center in Bhaktapur from January 2022 to December 2022. The ethical clearance was obtained from Institutional review committee of Tilganga Institute of Ophthalmology (Reference no 27/2021). Detailed history and ocular examinations along with Schirmer test and Tear Film Break Up Time were performed.

**Results:**

A total of 384 postmenopausal women were enrolled in the study. 276 (71.88%) patients had dry eye disease.100 (36.23%) patients belonged to 60-69 years of age group. 69 (25%) patients had mild dry eye, 112 (40.58%) patients had moderate dry eye and 95 (34.42%) patients had severe dry eye. Among 235 women with >10 years duration of menopause, 182 (77.45%) patients had dry eye.

**Conclusions:**

Dry eye is the common disorder that occurs in postmenopausal women, that is highly undiagnosed. The increase in age and duration of menopause are related to dry eye.

## INTRODUCTION

Dry eye is a multifactorial disease of tear film & ocular surface resulting in symptoms of ocular discomfort, visual disturbance and tear film instability with potential damage to the ocular surface associated with hyperosmolarity of the tear film and inflammation of ocular surface, as defined by International Dry Eye Workshop (DEWS) in 2007.^1^ Dry eye can lead to potential blinding infections or sometimes be a manifestation of life threatening systemic condition.^2^ Large epidemiological studies documented the prevalence of dry eye disease (DED) from five to over 30%.^3,4^ In general it was reported that the prevalence of dry eye disease was more prevalent in Asian countries compared to Western countries.^5^ Epidemiologic studies showed it is more prevalent among women (particularly post-menopausal) and elderly population.^6,7^ Studies have demonstrated that there is a hormonal etiology behind this group’s susceptibility to DED.^8-10^ The main aim of this study is to evaluate prevalence of dry eye and assess its relation to age of post-menopausal women.

## METHODS

It was cross sectional study done in a community eye center in Bhaktapur from January 2022 to December 2022. The ethical clearance was obtained from Institutional Review Committee of Tilganga Institute of Ophthalmology (Ref no:27/2021). Sample size was calculated using the formula

n= (Z^2^ × p × q) / e^2^

 = (1.96^2^ × 0.52 × 0.48) / 0.05^2^

Where, n= minimum required sample size Z= 1.96 at 95% Confidence Interval (CI) p= prevalence of dry eye in port menopausal women is 52%^11^ q= 1-p, e=margin of error 5%

Assuming the prevalence rate of 52%,^11^ i.e. p = 0.52, q=0.48, Z= 1.96, error be 5% i.e. 0.05, the minimum sample size for the study calculated was 384. Every alternate postmenopausal female visiting the Out Patient department was taken. Written informed consent was taken from all patients who participated in the study. Confidentiality was maintained. All post menopausal cases defined as 12 months after the final period visiting the hospital and gave consent to the study were included. Patients with ocular inflammation, history of ocular trauma, under topical and systemic medications that can cause dry eye, any systemic diseases associated with dry eye like diabetes, rheumatoid arthritis, thyroid disease, other autoimmune diseases, hormonal therapy and eyes that have undergone recent ocular surgeries were excluded. All cases fulfilling the criteria underwent detailed history taking in predesigned format. Patient was asked for ocular symptoms of dry eye like redness of eye, grittiness, foreign body sensation, burning sensation, dryness, crusting of eyelashes, eye fatigue, feeling of dryness in eyes, ocular discomfort, watering and itching.

Ocular examination including visual acuity, slit lamp examination for lid margin swelling over lid margin, meibomian gland dysfunction, bitot’s spots, conjunctival or ciliary congestion, conjunctival discharge, corneal vascularization, anterior chamber, iris, pupil, lens was done and biomicroscopy was performed. Dry eye tests schirmer’s test and tear film break up time (TBUT) were done and severity of dry eye was classified accordingly. Special standardized filter paper strips were placed inside the lower lid of both eyes. Subjects were asked to close their eyes for five minutes and the paper was removed after five minutes. The amount of moisture on the filter strips was noted immediately. In order to measure only the basal secretion of tears, anesthetic proparacaine hydrochloride eye drops were administered five minutes prior to testing. The value of <5mm is considered as severe dry eye, 5-8mm is moderate dry eye, 9-14 mm is mild dry eye and >15mm is normal.^11^ The tear film break up time describes the stability of the tear film. Normal values are between 20 to 30 seconds. Values less than 10 seconds are pathological.^30^ Data entry, cleaning and coding were done in Microsoft excel. Statistical analysis was done in Statistical Package for the Social Sciences (SPSS) Statistics version 20.0 (IBM Corp., Armonk, NY, USA). Descriptive statistics such as mean, standard deviation (SD), frequencies, percentages were determined.

## RESULTS

Among the total enrolled 384 cases, the average age was 64.06±9.78 years, range 41-96 years. Among the total participants, 276 (71.88%) had dry eye disease and 108 (28.10%) patients had normal eye (Table 1).

Symptoms of dry eye was present in 195 (70.65%) patients whereas 81 (29.34%) patients had subclinical dry eye. Among 138 postmenopausal women in 60-69 years age group, 100 (72.46.%) patients had dry eye and among 123 women over 70 years, 95 (77.24%) patients had dry eye (Table 2).

**Table 1:** Prevalence of dry eye among postmenopausal women(n=388).

	n(%)	Mean ± SD
Dry eye	276(71.88)	64.73±9.61
Normal	108(28.10)	62.37±10.07

**Table 2:** Distribution of dry eye according to age (n=388).

Age group	No. of patients	Patients without dry eye n(%)	Patients with dry eye n(%)
40-49	13	5(38.46)	8(61.54)
50-59	110	37(33.64)	73(66.36)
60-69	138	38(27.54)	100(72.46. )
>70	123	28(22.76)	95(77.24)

**Table 3:** Distribution of patients on the basis of dry eye severity (on the basis of Schirmer’s test after anesthesia) (n=276).

Severity	n(%)
Mild (9-14 mm)	69(25.00)
Moderate (5-8 mm)	112(40.58)
Severe (<5 mm)	95(34.42)

**Table 4:** Indoor and Outdoor Residence in Relation to Dry Eye Status (n=384).

	Dry eye n(%)	Normal n(%)
Indoor	47(63.51)	27(36.49%)
Outdoor	229(73.87)	81(26.12%)

**Table 5:** Distribution of Dry Eye According to Duration of Menopause (n=384).

Menopause duration	Number of patients with dry eye n(%)	Number of patients without dry eye n(%)
Upto 5	55(61.11)	35(38.89)
5-10 years	39(60.10)	20(33.90)
>10 years	182(77.45)	53 (22.55)

Out of 276 patients with dry eye, 69 (25%) patients had mild dry eye, 112 (40.58%) postmenopausal women had moderate dry eye and 95 (34.42%) postmenopausal women had severe dry eye (Table 3).

TBUT <5 seconds was seen in 81 (29.63%) patients and <10 seconds in 145 (52.57%) patients out of 276 subjects. Among 310 postmenopausal women with outdoor exposure related to occupation, 229 (73.87%) patients had dry eye while among 74 postmenopausal women with indoor related occupation, 47 (63.51%) patients had dry eye (Table 4).

Among 235 women with >10 years duration of menopause, 182 (77.45%) patients had dry eye while among 90 women with upto 5 years duration of menopausa, 55 (61.11%) had dry eye (Table 5).

## DISCUSSION

Hyperosmolarity can result in symptoms of ocular discomfort, dryness and vision disturbances. There are mainly two types of dry eye namely aqueous deficient dry eye and evaporative dry eye. Aqueous deficient dry eye is defined by the presence of normal evaporation with a reduced tear volume and is associated with lacrimal gland dysfunction. Evaporative dry eye is characterized by a normal tear volume with an increased rate of tear evaporation and is usually associated with meibomian gland dysfunction. Dry eye is not a disease entity, but a symptom complex which occurs due to deficiency or abnormalities of tear film. Tear film helps to maintain a normal healthy ocular surface as well as providing a smooth refractive surface for optimal vision. A crucial factor in dry eye is the loss of tear film stability which leads to tear film hyperosmolarity. ^11^ Dry eye is one of the most commonly overlooked sign of menopause.

The aim of our study was to find out the prevalence of dry eye in post-menopausal women attending in our center and its association with increase in age. The present study showed 71.88% of post menopausal women visiting the out patient department services had dry eye disease with peak among 6069 years. This study showed the presence of dry eye disease in patients without symptoms in 81 (29.34%) patients. Therefore, even patients without dry eye symptoms can have dry eye which can easily be diagnosed with the Schirmer’s test. The prevalence of dry eye disease has been reported in many countries around the world, with a range of between 9.5-90%.^3,12-20^ Large scale epidemiological studies done in the United States have shown that the rate of DED in women over 50 years old is nearly double than that in men over 50, at 7% and 4%, respectively. ^21^ Postmenopausal women are more commonly affected by dry eye disorders.^22^. In our study, we have found that the prevalence of mild dry eye in 34.42%, moderate in 40.58% and severe dry eye in 25.00% patients. This is similar to the study done in Biratnagar that showed 66% of postmenopausal women had dry eye, 21% mild, 26% moderate and 19% severe dry eye with the mean age of 59.9 years.^23^ A study done in Karnataka revealed dry eye in 66.6% of post-menopausal women with majority in the age group being between 65 to 75 years (27.7%).^24^ A study done in postmenopausal women in North India also showed prevalence of dry eye in 73.33% of postmenopausal women commonly manifested in 55-59 years and 60-64 years age group with severe DED in 24.24%.^25^ The study done in medical college in Chennai showed the prevalence of dry eye in 52% women and majority were in the age group of 55-59 years, mild dry eye (27.5%) followed by moderate dry eye in 21%.^11^ Study by Agarwal R showed dry eye prevalence rate of 32%, 21% had mild, 8% moderate and 3% severe dry eyes.^26^ Studies conducted in Madhya Pradesh and Chennai showed slight low prevalence in dry eye compared to my study conducted in Bhaktapur city. The reason might be the moderately humid climate in former compared to dry dusty winter and colder temperatures in Bhaktapur which increases more tear evaporation and more dry eye. Many households in Bhaktapur still use biomass fuels like wood, dung, crop residues for cooking which can lead to more smoke exposure and Bhaktapur populations are also more exposed to traffic and industrial pollution. Thus, the mild variation in prevalence in dry eye could be due to the geographical variation and environmental diversity.

The dry eye was seen in 73.87% among total outdoor post menopausal group compared to 63.51% on total group residing indoor, similar to other studies.^2,29^ This can be explained by more exposure of outdoor workers to environmental irritants like smoke, dust and sunlight.

Different studies indicated that old age and female sex are^27,28^ established risk factors of dry eye disease. The effect of hormones on the incidence and course of the dry eye has been noted, in particular in postmenopausal women.^29^ This study showed that dry eye becomes more prevalent with age due to decreased tear production and changes in tear film composition. Similar findings were seen in other studies.^11,25^ In the present study, 94 (34.05%) cases with postmenopausal duration of < 10 years and 182 (65.94%) cases with post menopausal duration > 10 years had dry eye which was similar to other studies.^24-26^

Dry eye in post-menopausal women is evolving issue and has gained much importance in the recent years as the dry eye is more common and once the disease evolves it is difficult to treat due to physiological hormonal changes during menopause and poor compliance of these patients in this age group. ^25^

Various mechanisms such as decreased tear production or increased evaporation and changes in receptor receptivity interplay to alter the ocular surface homeostasis, decreased in hormonal levels and subsequently result in DED. Several studies have suggested potential role of hormone replacement therapy in menopause-associated dry eye symptoms.^30^

The drawback of this study was lack of lissamine green dye use to access dry eye severity which stains damaged or dead cells on the ocular surface to identify area of inflammation and epithelial cell compromise. Also, the study did not involve the study of sex hormones assay, since various studies have shown that the reduction of estrogens level may lead to dry eye.

## CONCLUSIONS

Dry eye is the common disorder that occurs in postmenopausal women, that is highly undiagnosed. The increase in age and duration of menopause are related to dry eye.

## Data Availability

The data are available from the corresponding author upon reasonable request.
